# Lifestyle Modifications to Help Prevent Headache at a Developmental Age

**DOI:** 10.3389/fneur.2020.618375

**Published:** 2021-02-02

**Authors:** Umberto Raucci, Alessandra Boni, Melania Evangelisti, Nicoletta Della Vecchia, Margherita Velardi, Fabiana Ursitti, Gianluca Terrin, Giovanni Di Nardo, Antonino Reale, Alberto Villani, Pasquale Parisi

**Affiliations:** ^1^Pediatric Emergency Department, Bambino Gesù Children's Hospital, Institute for Research, Hospitalization and Health Care (IRCCS), Rome, Italy; ^2^Department of Pediatrics, Sapienza University, Rome, Italy; ^3^Department of Pediatrics, Department of Neuroscience, Mental Health & Sense Organs (NESMOS), Faculty of Medicine & Psychology, c/o Sant'Andrea Hospital, Sapienza University, Rome, Italy; ^4^Division of Neurology, Bambino Gesù Children's Hospital, Institute for Research, Hospitalization and Health Care (IRCCS), Rome, Italy; ^5^Department of Gynecological Obstetric and Urological Sciences, Faculty of Medicine and Dentistry, Sapienza University of Rome, Rome, Italy

**Keywords:** headache, migraine, tension-type headache, lifestyle, risk factors, pediatric, quality of life

## Abstract

Headache is the world's seventh most significant cause of disability-adjusted-life in people aged between 10 and 14 years. Therapeutic management is based on pharmacological approaches and lifestyle recommendations. Many studies show associations between each migraine-promoting lifestyle, behavioral triggers, frequency, and intensity of headaches. Nevertheless, the overall aspects of this topic lack any definitive evidence. Educational programs advise that pediatric patients who suffer from migraines follow a correct lifestyle and that this is of the utmost importance in childhood, as it will improve quality of life and assist adult patients in avoiding headache chronicity, increasing general well-being. These data are important due to the scarcity of scientific evidence on drug therapy for prophylaxis during the developmental age. The “lifestyle recommendations” described in the literature include a perfect balance between regular sleep and meal, adequate hydration, limited consumption of caffeine, tobacco, and alcohol, regular physical activity to avoid being overweight as well as any other elements causing stress. The ketogenic diet is a possible new therapeutic strategy for the control of headache in adults, however, the possible role of dietary factors requires more specific studies among children and adolescents. Educational programs advise that the improvement of lifestyle as a central element in the management of pediatric headache will be of particular importance in the future to improve the quality of life of these patients and reduce the severity of cephalalgic episodes and increase their well-being in adulthood. The present review highlights how changes in different aspects of daily life may determine significant improvements in the management of headaches in people of developmental age.

## Introduction

Migraine and other headache syndromes represent an enormous source of morbidity, especially among the pediatric population. In a 2016 study, the Global Burden of Disease (GBD) indicated that migraine is the second leading cause of years lived with disability (1), up from the seventh cause in GBD 2010 (2). Particularly in adolescence, headache is a cause for common neurological conditions in Europe, especially in women, with an estimated average prevalence of 54.4% (3, 4). This represents the seventh most significant cause in the world for years of life adjusted for disabilities in subjects between 10 and 14 years (5).

Headache frequency is not better controlled with a pharmacological approach compared to placebo as demonstrated in The Childhood and Adolescent Migraine Prevention (CHAMP) study which matched the results of amitriptyline vs. topiramate vs. placebo for migraine prevention in 8 to 17-year-old patients and was stopped early for emptiness. This study hypothesized that active co-interventions, such as management of lifestyle, limiting the use of medications only to cases of acute attacks, may have contributed to the high placebo response rate. In the CHAMP study, the healthy habits prescribed to children and adolescents for the prevention of migraine attacks were: adequate hydration, regular exercise, avoidance of skipping meals, and maintaining regular sleep (6).

**Figure 1:**
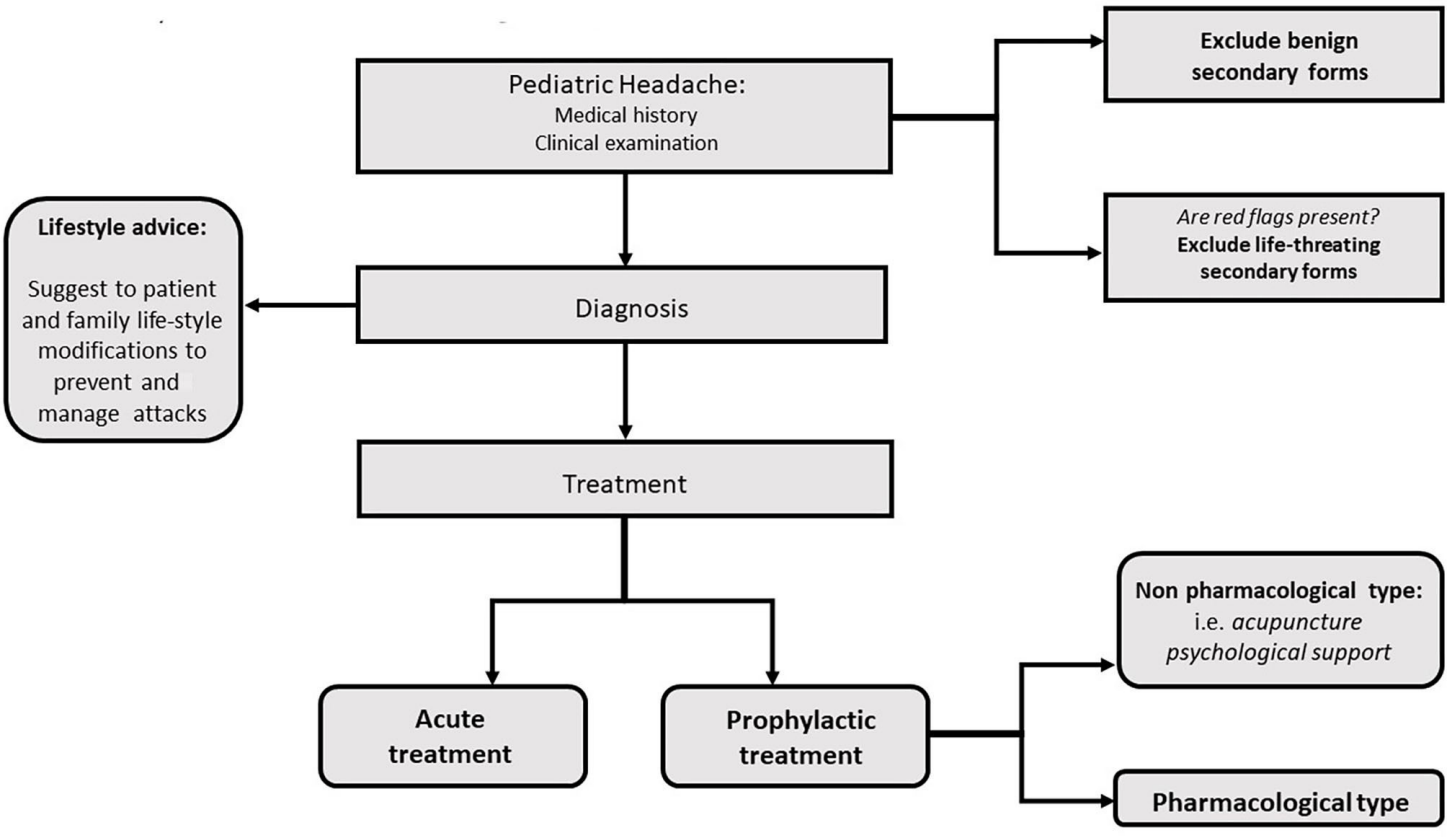
Lifestyle advise in headache management plan.

**Figure 2:**
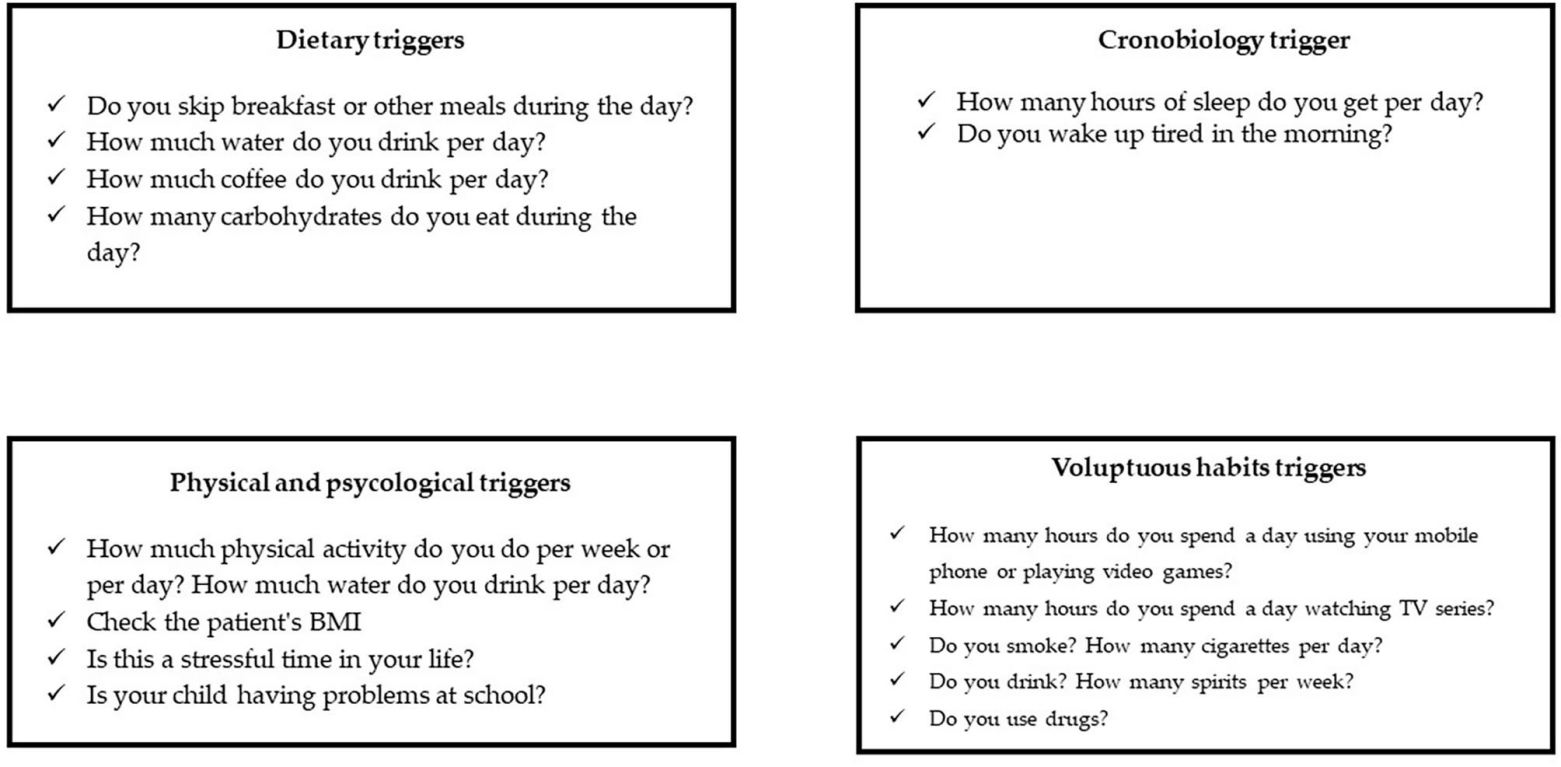
Lifestyle habits to be investigate in pediatric headache management.

Education about lifestyle factors is an important part of any headache management plan. Since headache management is also based on non-pharmacologic complementary and integrative treatments, healthy lifestyle habits should be a focus in the treatment approach (Figures 1, 2). Potentially modifiable lifestyle factors associated with headache are advocated. High consumption of caffeine, insufficient fluid intake, and irregular intake of meals were identified as contributing to trigger or prolong headache attacks or headache frequency; other lifestyle factors, such as physical inactivity, smoking, or excess of alcohol were also reported to be associated with headache in adults (7). Physical inactivity in pediatric age is known to be an important predictive factor for obesity in adulthood. In particular, recent data show an association between obesity and headache disorders also in children (8, 9); in fact, the same pathophysiological mechanisms appear to be implicated in obesity and migraine such as thalamic activation, and the release of serotonin as a neurotransmitter along with adiponectin as an immune modulator, suggesting that lifestyle and behavioral differences may contribute to the migraine-obesity connection (10).

In clinical practice, it may be observed the growing use of ketogenic diet (KD) on the hypothesis that KD should contribute to restore brain excitability and metabolism and counteract the neuroinflammation present in migraine, although its specific mechanism remains unclear (11). Meal skipping should also be linked to subjective somatic and psychological health complaints in a pediatric population, as shown in the CASPIAN-V study (12). Another lifestyle factor contributing as a “trigger” for headaches is stress. This is particularly true at adolescent age. Due to its physical, psychological, and biological changes, it is indeed considered a transitional and critical period.

The use of “life-style rules” in the management of pediatric headache is of vital importance as these strategies may also improve the quality of life in future adulthood, provided that educational promotion starts during the pediatric age. Many randomized, double-blind, and placebo-controlled trials have recently suggested that there is no significant decrease in headache frequency or headache-related disability in childhood and adolescent migraine when it is treated with the available drugs. In particular, no significant differences have been reported in pediatric studies when comparing pharmacotherapy with placebo (6, 12–14). Moreover, the active drugs are associated with higher rates of adverse events.

In pediatric age, these aspects should be taken into account more carefully than in adults, as the placebo response, in children, may have a more significant impact on the sample size under investigation.

Based on these pieces of evidence, pediatricians should help migraine-children in limiting the impact of lifestyle triggers, thereby mitigating the severity and frequency of their attacks by offering lifestyle programs. Sleep, exercise, eat/obesity, cognitive impairment, bullying, and other family and school-related stressors as well as other comorbidities may play a crucial role in this respect. This review highlights how at the developmental age, modifications of various aspects of daily life may lead to improving changes in headache. The search for the best pieces of evidence associated with each one of these factors may produce best-practice recommendations.

## Methods

Papers published up to August 2020 were selected through a computerized literature search using PubMed, ISI Web of Science databases. The following terms were entered, individually or in combinations: lifestyle, sleep, skipping meals, diet, breakfast, caffeine, hydration, overweight, obesity, smoking, alcohol, stress, physical activity, inactivity, technology, electronic device, ketogenic diet, children, pediatric, headache, and migraine/headache.

No restrictions were made on the publication date, study design, and language. A cross-reference search was carried out to identify any further relevant data.

We conducted a literature search and papers relevant to this review are included in the list of references.

## Headache and Lifestyle

### Adequate Hydration and Headache

According to reports and literature, the clinical practice lifestyle recommendations for migraine management include consistent intake of non-caffeinated fluid (on average 8–10 cups per day, adolescent population) (15). Nevertheless, to the best of our knowledge, no data are reported in the literature regarding the likelihood that an increased intake of water in children may help to decrease the frequency of headache in pediatric migraine. On the other hand, there is evidence, from studies on adults affected by migraine, that an increased water intake might decrease headache severity, frequency, and duration. A randomized clinical trial, conducted in a primary care setting among 102 adult patients evaluated through the Migraine-Specific Quality of Life (MSQOL) test, concluded that drinking more water determined a statistically significant improvement by 4.5 points on MSQOL. In addition, a 6 point improvement on a 10-point scale was demonstrated among patients who drank more water (in 47% of patients) compared with the control group.

According to these data, this non-invasive protocol for a short period of time could be a reasonable recommendation, at least to verify any improvements that result from an increase in water intake (16).

Kenney et al. (17) examined systematically the adherence to medication and lifestyle in adolescents with migraines and concluded that the population in the study did not drink enough non-caffeinated fluids each day. Indeed, most children and especially adolescents tend to be quite dehydrated. The study evaluated the hydration status of Unity States children and adolescents (4,134 participants, 6–19 years old) and calculated the mean urine osmolality and the relation to inadequate hydration (urine osmolality >800 mOsm/kg); the prevalence of inadequate hydration was 54.5% (17). A recent review of 32 studies also indicated that under-hydration was one of the main problems, suggesting that children are not drinking enough water to be adequately hydrated (18).

The observation that water deprivation can provoke migraine was recognized also by migraineurs; 34 of 95 (36%) adult subjects interviewed indicated that insufficient fluid intake is a factor that precipitates migraine (19).

Therefore, adequate hydration is important in the management of headache (Table 1). From the pathogenetic point of view, fluids can help increase blood volume, resulting in better oxygen delivery to the brain (20), and maintain the balance of cellular ion concentrations to ensure a correct plasma osmolality and sodium concentration (21, 22).

**Table 1:** Adequate hydratation.

		**Institute of medicine recommendations**		**European food safety authority**	
		**Total water intake**	**Total water intake**	**Total water intake**	**Total water intake**
		**(fluids + foods)**	**(only fluids)**	**(fluids + foods)**	**(only fluids)**
Gender	Boys	2.4 l/day	1.9 l/day	2.1 l/day	1.7 l/day
	Girls	2.1 l/day	1.7 l/day	1.9 l/day	1.5 l/day

*Modified by Suh and Kavouras (18)*.

### Overuse of Caffeine and Headache

Caffeine abuse among teens is an increasing practice in age-related behaviors and can lead to triggering or exacerbating headaches. Children (but especially adolescents) also drink large volumes of caffeine-containing carbonated drinks daily. Adolescent girls frequently utilize diet colas as substitutes for food, unaware of the risk of caffeine dependency (23). Approximately 55% of caffeine is consumed as a soft drink by young American schoolchildren (24).

Caffeine withdrawal has also been identified as a cause of headache by the International Headache Society (Table 2). However, excessive caffeine consumption, particularly in the form of coffee and tea, seems to be an as yet unrecognized cause of headache and migraine (25). Caffeine-withdrawal headache has been reported in the case of the customary daily consumption of as much caffeine as for about 1 cup of coffee per day (26).

**Table 2:** Headache attributed to a substance or its withdrawal.

**Headache attributed to use of or exposure to a substance**
Nitric oxide (NO) donor-induced headache
Phosphodiesterase (PDE) inhibitor-induced headache
Carbon monoxide (CO)-induced headache
Alcohol-induced headache
Cocaine-induced headache
Histamine-induced headache
Calcitonin gene-related peptide (CGRP)-induced headache
Headache attributed to exogenous acute pressor agent
Headache attributed to occasional use of non-headache medication
Headache attributed to long-term use of non-headache medication
Headache attributed to use of or exposure to other substance
**Medication-overuse headache (MOH)**
Ergotamine-overuse headache
Triptan-overuse headache
Non-opioid analgesic-overuse headache
Opioid-overuse headache
Combination-analgesic-overuse headache
Medication-overuse headache attributed to multiple drug classes not individually overused
Medication-overuse headache attributed to unspecified or unverified overuse of multiple drug classes
Medication-overuse headache attributed to other medication
**Headache attributed to substance withdrawal**
Caffeine-withdrawal headache
Opioid-withdrawal headache
Oestrozen-withdrawal headache
Headache attributed to withdrawal from chronic use of other substance

*Modified by Headache Classification Committee of the International Headache Society (IHS) (25)*.

Hering-Hanit and Gadot (27), described a correlation between the reduction in headache recurrence and the use of caffeine-drinks in a pediatric population. The study cohort was composed of 36 children and adolescents (17 girls and 9 boys), enrolled for 5 years, with daily or near-daily headaches related to excessive caffeine intake in the form of cola drinks. All enrolled subjects were heavy cola drinkers; at least 1.5 L of cola drinks per day (192.88 mg of caffeine per day) and an average of 11 (range 10.5–21) L of cola drinks per week, which equals 1414.5 mg of caffeine (range 1350.1–2700.3). The gradual withdrawal of cola drinks resulted in the complete cessation of all headaches in 33 subjects over a 24-week follow-up. In addition, the importance of creating lifestyle guidelines between parents and headache patients was advocated, showing that gradual withdrawal could have been achieved without withdrawal headache (27).

In the TEENs study, a cross-sectional observational study conducted in Spain on students (12–18 years old) from six different schools, headaches were significantly more frequent in people who overused caffeine (30.9 vs. 24.7%, *p* = 0.009). Moreover, caffeine consumption was particularly decisive in older adolescents (14–18 years old) (28).

In a large cross-sectional study among high-school students, elevated consumption of coffee was significantly associated with migraine and tension-type headache, with an about twice relative risk (OR = 2.4; 95% confidence interval 1.3–4.7). Markedly, migraine demonstrated a 3 times higher relative risk (OR = 3.4; 95% confidence interval 1.6–7.0) (7).

As reported in a population-based case/control study on an adult population (26), case-patients manifesting chronic daily headache (CDH) (by convention occurring ≥15 days/month), were more likely to have been high caffeine consumers before the onset of CDH (OR = 1.50, *p* = 0.05), compared with control-patients affected by episodic headache.

### Physical Activity and Headache

Physical activity, especially aerobics, may represent a non-pharmacologic option in headache management and can play a role in modulating pain processing: opioid, serotonin, and NMDA mechanisms acting in the rostral ventromedial medulla, promoting analgesia when associated with exercise (29). Exercise does not only reduce pain perception but also has a positive influence on conditions such as stress, depression, anxiety, and mental health, which are often associated with chronic pain.

Large population-based studies indicate that a reduction in physical activity and fitness level, as well as becoming overweight, are lifestyle-related risk factors for migraine and are associated with a higher prevalence and frequency of migraine (30–33). In school-aged children, subjects with migraine mostly prefer sedentary activities and less exercise in their leisure time, compared to the general population (34). A cross-sectional study demonstrated that adolescents with headaches have a sedentary life and exercise less frequently than teenagers who do not suffer from migraines (28).

Furthermore, some evidence states that, in patients with migraine, aerobic physical therapy reduces the number of migraine episodes, with an associated tendency to decrease pain intensity and the duration of migraine attacks (35–38).

Evers S. demonstrated in 2008 that Topiramate is both effective and well-tolerated in preventing migraine, and was elected as a first choice in adult prophylactic treatment (39). Varkey et al. (40) proposed a randomized controlled trial to study in migraine adult patients, the effect of prophylactic use of topiramate compared with non-pharmacological prophylactic treatments, such as relaxation and aerobic physical exercise. The absence of a significant difference in the number of episodes between the two groups highlights the efficacy of non-pharmacological prophylactic treatments and supported the opinion that exercise may be an option in preventing migraine attacks in patients who do not benefit from (or do not want to take) daily medication.

Although, in the long run, physical activity can help prevent migraines, acute phase biological effects can be triggered by physical exercise, some of which could have a role in the pathogenesis of migraine. Koppen et al. (41) interviewed a group of people who suffer from regular migraines and concluded that 38% of them reported exercise-triggered attacks, so much to, that more than half of them had to quit the sports activity as a result. Varkey et al. (42) showed that maximal aerobic exercise might trigger a migraine, but it does not always provoke an attack, even in the migraineur population who suffer from exercise-related migraines. The individuals who consistently experience physical activity-related pain worsening may require tailored interventions to gradually increase exercise to reach a chronic adaptation and benefit from the other long-term advantages of a more active lifestyle.

Hanssen et al. (43) compared a mild continuous training program with an elevated intensity interval training program in a group of migraineurs. After a 12-week intervention period, both exercises appeared to be effective in reducing migraine days, although an intensive interval training program seemed more effective than continuous training.

In light of the aforementioned evidence, to obtain the most advantageous result, migraine patients should be encouraged to use physical activity with cautiously increased modalities (in terms of intensity, frequency, and duration), adapting the training pattern to the individual patient.

### Ketogenic Diet and Headache

The ketogenic diet consists of a dietary regimen that induces and maintains a basal state of ketosis. In this metabolic condition ketone bodies are used as an energy source for the body. Ketone bodies include three compounds, such as acetone, acetoacetic acid, and beta-hydroxybutyric acid, and are present in the blood in small quantities.

Fasting is the first condition in which the production of ketone bodies is activated. Low-calorie or particularly high-fat diets have the same result of activating the production of ketone bodies.

The ketogenic diet has been used for years to treat various clinical conditions in both the adult and pediatric population, including pharmaco-resistant epilepsy. Recent studies, including randomized trials, are applying this dietary regimen in adult patients with chronic migraine.

The underlying mechanisms of action of the ketogenic diet in these neurological conditions are currently being investigated. The hypothesized and supported mechanisms were recently described as follows: (1) Reduction of glycolysis and increase of lipid oxidation with the supply of substrates for energy production in the Krebs cycle; (2) Mitochondrial biogenesis and increase in brain energy reserves; (3) Alteration of brain neurotransmitters with increased levels of gamma-aminobutyric acid (GABA) and inhibition of glutamatergic synaptic transmission; (4) Activation of cytoplasmic or membrane ATP-dependent potassium channels; (5) Neuroprotective effects through an increase in energy reserves and a reduction in the production of free radicals; (6) Positive effects against cortical spreading depression; (7) Reduction of the inflammation generated by macrophages; (8) Alteration of the microbiome and its beneficial effects on intestinal permeability, the synthesis of metabolites and neuropeptides (44).

Few studies reported in the literature have demonstrated the efficacy of the ketogenic diet in adult patients with migraine, while rare cases have been reported in pediatric age.

Strahlman was one of the first authors to report on the efficacy of the ketogenic diet as a migraine prophylaxis approach. In 2006 he reported on a woman (his wife) who had been suffering for years from chronic migraine attacks treated with different prophylactic drugs, in which the ketogenic diet seemed to have resolved migraine (45).

Kossoff et al. (46) were among the first in 2010 to run a prospective, open-label study that included adolescents aged 12–19 years with chronic migraine treated with the modified Atkins diet (MAD). Eight patients were enrolled for 2 years (three males and five females, aged 13–16 years). Three patients completed the 3 months period of the study, while the remaining five completed the study before week 6 due to the absence of effects and poor adherence to the diet. The results of this study were not favorable for the use of the ketogenic diet in migraine. No patient showed a reduction in the frequency of migraine attacks. Only 2 patients, male and younger, reported an improvement in the intensity of the attacks. In any case, it is interesting to observe that this study represents the first published prospective study on dietary treatment for migraine in a pediatric population (46).

In another report in 2010, Urbizu et al. described the cases of two monochorionic twins who, between 5 and 10 years of age, developed a mixed neurological condition consisting of exercise-induced paroxysmal dyskinesia, migraine with and without aura, absence seizures, and cramping pains while writing. Genetic investigations documented a *de novo* heterozygous missense mutation in exon 4 of the SLC2A1 gene, associated with the GLUT1 deficiency syndrome. It is interesting to note that both twins responded positively to the ketogenic diet. Therefore, this study confirmed that in the intermediate form of the clinical spectrum of GLUT1 deficiency, the ketogenic diet remains a cornerstone from a therapeutic point of view, although not specific for migraine (47).

In 2013, Di Lorenzo et al. (48) reported cases of two 47 year old twin sisters suffering from high-frequency migraines, whose migraine attacks were reduced by a ketogenic weight loss diet. This accidental and retrospective observation led to the hypothesis that a ketogenic diet could be useful for patients with migraine (48).

In contrast, Bond et al. (49) proposed that weight loss could improve migraines in obese women. Despite several other factors, such as the reduction of analgesia, restraint from trigger foods, and nutrient intake could have contributed to the unexpected outcome observed in the twin sisters, and the authors concluded that KD improved migraine. The first observation documented that migraine had improved before the final weight loss. In addition, the withdrawal of KD has been linked with the recurrence of migraine, despite the persistence of body weight. The second observation demonstrated that nutraceuticals do not play a role in migraine improvement, since they must have been taken at higher concentrations and for a longer time to induce some effects. Finally, triggers for migraine, such as phenylethylamine, tyramine, aspartame, monosodium glutamate, nitrates, and nitrites were not avoided during KD. Consequently, the observed improvement was due more probably to ketogenesis. The authors hypothesized that the improvement of migraine may have been explained with the dampening of inflammation and modified neuro-inflammatory phenomena, the modulation of cortical excitability, the inhibition of oxidative stress in neurons, the depression of the phenomenon of cortical diffusion, together with the activation of cerebral mitochondrial metabolism. However, these cases provided only class IV evidence, as the authors' hypothesis that KD weight loss may lead to an improvement in migraine was based only on retrospective observation of a small number of cases (48).

Later in 2015, Di Lorenzo et al. (50) reported that the mechanisms underlying the effectiveness of KD could be related to its ability to improve mitochondrial energy metabolism and counteract neural inflammation. In this study, the authors included overweight female patients with migraine (96 patients in total) and examined whether the ketogenic diet could improve headache characteristics regardless of weight loss. Their most innovative result was that while on the ketogenic diet, patients experienced a significant reduction in the number of headache days, frequency of attacks, and medication intake for the attack per month, compared to the baseline period (50).

In 2016, Di Lorenzo et al. (51) showed that characteristics of migraine had improved during 1 month of the ketogenic diet, in terms of frequency and duration, confirming their previous clinical findings. The most surprising discovery of this study was that KD significantly normalized the addiction deficit on evoked potentials, provided that the clinical features of migraine improved. Various mechanisms, such as the induction of neural plasticity, changes in cortical excitability, and increased energy metabolism, could explain these results. The results of this study suggested that KD selectively affected delayed habituation by reducing cortical hyperreactivity in migraineurs. Both the increase in rapid GABAergic neurotransmission (GABAA) and decrease in the probability of release of excitatory neurotransmitters from presynaptic neurons have been implicated in the anticonvulsant actions of KD. Considering that the brain of migraineurs is in an imbalance between arousal and inhibition in sensory cortices, it is very interesting that KD, as reported, plays a role in increasing and maintaining synaptosomal GABA content at a higher value, in increasing intracellular concentration, and in causing a presynaptic reduction in glutamate input (51).

In a subsequent study in 2019, Di Lorenzo et al. (52) reported that the positive clinical effects observed in a population of migraineurs with 1-month of KD treatment coexisted with a normalization of the typical addictive inter-ictal deficit at the cortical level, although not in the brainstem. These results suggest that the cerebral cortex could have been the primary site of KD-related modulation (52).

Yet again, in 2019, Di Lorenzo et al. (53) confirmed, in a double-blind study, that VLCKD (Very Low Calorie Ketogenic Diet) was effective for a rapid and short-term improvement of migraine in overweight patients in contrast to VLCnKD (Very Low Calorie non-Ketogenic Diet). The duration of this dietary strategy and the selection of overweight patients with migraine should be determined in future studies (53).

Recently, Gross et al. launched a study on the efficacy and safety of the use of ketone bodies as a prophylactic treatment for adult migraine patients, which consists of a randomized, placebo-controlled, double-blind, crossover study (54).

Finally, in a recent study by Moavero et al. (55), the efficacy and safety of KD in pediatric patients with chronic migraine was evaluated. In this study, patients (10–18 years of age) with chronic migraine, who did not respond to previous prophylactic treatments or refused other therapies, were enrolled. As a first step, the patients underwent a biochemical screening to rule out inborn errors of metabolism. KD was started in a 1:1 ratio. Sixteen patients, 3 males, and 13 females were enrolled to initiate KD. Among them, 8 decided not to start KD, so reducing the number of patients enrolled in the KD group to 8 patients, 1 male, and 7 female, with ages ranging from 11 to 18 years. In this study, the difficulties found in enrolling patients for KD were primarily due to the palatability of the diet itself. In the enrolled patients, partial efficacy was observed in 50% of cases. However, the benefits were transient in 2 of them. These final results were not considered sufficiently encouraging to justify such a diet in these patients (55). Although expressing a potential efficacy in treating chronic migraine, KD involved significant stress for both patients and parents and appeared to be a difficult treatment option during childhood and adolescence. Whereas, KD is increasingly used in adults affected by migraines, to date, data on efficacy in childhood and adolescence are still lacking.

#### Overweight and Headache

In pediatric ages and adulthood, migraine and overweight/obesity are common conditions with negative impacts on the quality of life. Due to the increased prevalence of overweight and obesity in pediatric patients over the last decade, the occurrence of migraine in the pediatric population has increased as well. Between 1980 and 2013 the prevalence of overweight or obesity in pediatric age children was 23,8% in boys and 22.6% in girls in developed countries, while it settled at 8.1–12.9% among boys and 8.4–13.4% among girls in developing countries (56).

Most interestingly, in recent years many authors have analyzed the association between obesity and migraine in adulthood and pediatric age patients (8, 9). Robberstad et al. (30) conducted a cross-sectional lifestyle analysis of 5,847 Norwegian adolescents, in which students were interviewed about headaches. The results showed that the relative risk of migraine was two times higher in overweight or obese subjects (OR 1.6; 95% CI 1.4–2.2, *p* < 0.0001). Furthermore, the recurrence of headaches in adolescent age appeared to be linked to being overweight, smoking, and sedentary lifestyles, whether the items were analyzed as independent factors or in combination (30). Ravid, in a retrospective study on 181 children evaluated because of headache, concluded that a higher prevalence (39.8%) of obesity occurred in pediatric groups relative to the general population. Unlike the tension-type headache, the diagnosis of migraine was undoubtedly associated with an increased risk of being overweight (OR = 2.37, *p* = 0.01) or overweight (OR = 2.29, *p* = 0.04). A significant independent risk for being overweight was present in females with migraine (OR = 4.93, *p* = 0.006). Disregarding the type of headache, a high body mass index percentile was associated with increased headache frequency and disability, except for the duration of attacks (57).

Several studies have reported that being overweight has a specific role in headache severity and the conversion from episodic to chronic migraine (58–60). Lu et al. (60) reported a more than doubled risk for chronic migraine in obese patients. Comparable results were observed in the Kinik et al. study (61) on 77 females (10–16 years of age). It has been also reported that obese patients have higher rates of headache frequency (considering both migraine and tension-type headaches) and intensity with more nausea and more missed school days (62).

Once more, as described by different reports, there is clear evidence of a beneficial effect of weight loss on headache severity (49, 63–65).

Considering that some authors did not confirm the association between overweight and migraine (66, 67), it is nevertheless necessary to implement research data to achieve more conclusive results.

It seems to be likely that the association between obesity and migraine in pediatric age is a multifactorial event involving both central and peripheral pathways (9). Currently, the role played in migraine attacks by the hypothalamus and the bioactive neurotransmitters and neuropeptides modulating the energy homeostasis, namely serotonin, orexin, and adiponectins (68) are current subjects of focus.

#### No Breakfast and Headache

Diet plays an important role in headache: fasting meals, eating high-sugar foods, dieting too rigorously, and skipping meals could act as a trigger or make children and adults more likely to complain of headache (69–72). Headache brought on by skipping meals is often quite severe and accompanied by mild nausea. Premonitory signs of headache, such as yawning, pallor, sweating, a craving for sweet things, and mood changes are similar to the symptoms manifested when skipping meals.

Several dietary habits may be associated with headaches or chronicity.

Milde-Busch et al. studied the association between diet, lifestyle factors, and different types of headache among a total of 1,260 adolescents. They used questionnaires to evaluate the intake of meals, coffee, non-alcoholic, and alcoholic drinks, the event of smoking, and the quality of physical activity undertaken. They found no correlation between different types and skipping meals (7).

An Italian population-based study among pre-adolescent and adolescent students reported that 365 of over 800 subjects complained about headache episodes (45, 6%) associated with anorexia. In more than 50% of cases, the event caused the patients to miss school. A strict association between the headache, irregular intake of meals (especially irregular breakfast), and sleep disturbance were also documented (73).

The preliminary results of a population-based study on preadolescent/adolescent students (800, divided into two groups: with headache and without headache) showed that headache patients were mostly women (*P* = 0.006), and were irregular meal consumers (*P* < 0.0001), particularly breakfast (*P* < 0.0001). More frequent and painful attacks were reported among the population who skipped more often the meals (74).

On the basis of the results of a cross-sectional study (based on an anonymous questionnaire on demographic, lifestyle, medical data, presence of headaches and its features) and administered to 1,619 Spanish adolescents from six different schools, Torres-Ferrus et al. (28), reported that 30.5% of the subjects suffered recurrent headaches, of which 11.3% manifested migraine features. Univariate analysis of the results demonstrated, with a statistically significant difference, that headaches were more common in girls, teenagers, were significantly associated with a sedentary life, with the habit of skipping breakfast, smoking, and overusing caffeine.

The results reported are of clear importance to the design of educational programs and to improving the clinical practice for children and more specifically, adolescents.

### Technology and Headache

The exposure of children to technological devices, in terms of time spent using smart-phones, watching television, or playing videogames, has increased enormously during the last few decades, concurrent with a shortened engagement in physical activities. A large epidemiological study (involving 1,501,914 subjects) showed that 63% of adolescents (11–15 years of age) use mobile phones and, among them, 23% suffered from migraine, and 5% suffer from insomnia (that is known as strictly connected to migraine) (75). In a single-center, cross-sectional study involving a total of 123 patients, Demir and Sumer observed that the use of smartphones increases headache duration and frequency of migraine among adult patients due to ensuing poor sleep quality and daytime sleepiness (76).

The prevalence of 15 predefined trigger factors was considered in a retrospective descriptive study among 102 children and adolescents. Videogame overuse was reported as the fourth most common trigger referred by children with migraine (77).

Xavier et al. (78) reported a prevalence of 80.6% headache episodes in adolescents and its association with excessive use of electronic devices and games (OR 1,21). In terms of classification, 17.9% of cases were tension-type headaches, 19.3% were migraines, and 43.4% other types of headaches. Adolescents between 14 and 16 years were less likely (OR ≤ 0.68) to report the tension-type headache and other types of headache. The excessive use of digital equipment, electronic games, or attending the third year of high school proved to be risk factors for the development of migraine-type (OR ≥ 1.84) (78).

An epidemiological study on 11-year-old children failed to demonstrate that watching television was a trigger to improve migraine or tension-type headache (79) while a positive link between television watching combined with obesity and depression was proven in children and adolescents (80).

More detailed and long-term studies need to be implemented to explore the harmful effects of smartphone and videogame overuse, as well as an excess of time spent watching television, and to prevent effects on health.

### Regular Sleep and Headache

Sleep and headache are related by common neuroanatomical and neurophysiological substrates (81, 82). Studies necessarily rule out headache episodes that occur during sleep, those that are secondary to intracranial hypertension, or other life-threatening conditions.

“Sleep hygiene” refers to programs based on environmental conditions and practices, promoting continuous and restful sleep and including recommendations aimed at improving sleep habits. The programs advocate regularity of bedtime and waking time, encourage adequate time spent in bed for sustained and individually suitable sleep, endorse the restriction of beverages, foods, and the intake before bedtime of any substance able to disrupt sleep. Conversely, exercise, nutrition, and environmental factors enhancing sleep should be implemented to encourage “good sleep quality” (83), since sleep alterations (excessive, reduced or disrupted, deep sleep increase) may facilitate the onset of headache and migraine attack (84, 85).

To promote optimal health, the American Academy of Sleep Medicine recommends 9–12 h of sleep every 24 h for children aged 6–12 years of age, while teenagers 13–18 years of age should sleep 8–10 h per 24 h (86).

Due to the close relationship between regular sleep habits and headache in children and adolescents, several features of sleep are associated with episodes of headache (Table 3) Torres et al., in a survey dealing with adolescents, reported that 69.3% had regular sleeping habits (7.7 ± 1.1 mean sleep hours a day), while only 30.3% rated their sleep as restful (28). Specifically, students with headaches had shorter sleep time (7.6 ± 1.1 vs. 7.9 ± 1.1 h, *p* < 0.01) and reported non-regular sleeping habits, insomnia, daytime sleepiness, and unrestful sleep (*p* < 0.001), even when the data were adjusted by sleep time (28).

**Table 3:** Relationship between sleep and headache in children and adolescents.

1. Sleep could be a trigger factor for headache (excessive, reduced, or disrupted)
2. Sleep is often used by the patient to relieve headache
3. Bad sleep hygiene can worsen a pre-existing headache
4. Headache can be related to specific sleep stages (REM or SWS)
5. Headache occurs mostly during sleep or just after sleep; the association between headache and sleep is mediated by the same neurotransmitters (serotonin/dopamine)
6. Sleep disorders are often present in headache patients (restless legs syndrome, periodic limb movements during sleep, parasomnias, sleep-disordered breathing, narcolepsy)

*REM, Rapid Eye Movement; SWS, Slow Wave Sleep*.

Teenagers manifest variable sleep timing, chronically insufficient sleep, excessive daytime sleepiness, deficits in mood stability, learning ability, and control of impulsiveness. All these phenomena could be referred to as a delayed sleep phase and resulting from the negative consequences of the adolescent lifestyles on sleep bio-regulatory changes (87, 88).

Insomnia and sleeping <8 h are significant predictors for headaches in children and adolescents (28, 89).

Sleep state has been related to the occurrence of headache syndromes, and headache patients are more prone to sleep disorders (81, 83).

Headaches may occur during sleep, after sleep, and in relation to different sleep stages. Lack of sleep and excessive sleep are both considered possible triggers for headaches in children and adults. Indeed, especially in children, sleep constitutes the decisive factor for the resolution of a migraine attack (84, 90).

Both sleep and headache disorders are frequent health problems during childhood: the weighted aggregate migraine headache rate in children is 10.1%, while about as much as 25% of children had at least one type of sleep problem (81, 91, 92). Literature reports that children with headache usually manifest a higher rate of sleep difficulties, including co-sleeping, insufficient sleep, anxiety related to sleep, difficulties falling asleep, restless sleep, night waking, nightmares, and fatigue during the day (81, 93, 94). Different surveys based on large pediatric populations include the symptoms associated with headaches, such as parasomnias, insomnia, sleep-breathing disorders, and daytime sleepiness (81, 95–97).

As demonstrated by Roth-Isigkeit in a large population study (based on 662 children and adolescents), pain is strictly associated with sleep disturbances (53.6%), an inability to pursue hobbies (53.3%), eating problems (51.1%), and skipping school (48.8%). It is interesting to note that 60% of the population suffered from headaches (98).

A preferential occurrence of headache during REM sleep has been demonstrated. This observation could be explained by the silencing of the anti-nociceptive network of the periaqueductal gray matter (PAG), locus ceruleus, and dorsal raphe nucleus during REM sleep. Headaches triggered by sleep disorders can be classified as:

1. Headaches associated with obstructive sleep apnea (OSA), which include cluster headache, hypnic headache, and headache related to OSA.
2. Headaches related to insomnia, medication overuse, and psychiatric co-morbidity, including chronic migraine and tension-type headaches (90, 99).

Armoni et al. observed that children who were referred to the Sleep Clinic because of migraine episodes reported various sleep problems: in 40% of cases obstructive sleep apnea (OSA); in 27% of cases, insomnia; in 15% of cases periodic limb movement disorder (PLMD). Finally, 6 % of cases complained of hypersomnia (a Central Nervous System disorder). As for sleep patterns, children with migraine had significantly higher NREM 2 phase (*p* < 0.001) and a lower percentage of NREM3 phase (*p* < 0.001) compared to controls (after adjustment for demographics and the presence of sleep-disordered breathing) (100).

A strong link between cluster headache and hypnic headache, with the involvement of the hypothalamus, emerges from other literature. During REM sleep, a preferential presence of cluster headache, hypnic headache, and paroxysmal migraine may be observed. These preferential patterns could be explained by the silencing of the anti-nociceptive network of periaqueductal gray matter (PAG), locus ceruleus, and dorsal raphe nucleus during REM sleep.

The idiopathic hypnic headache usually occurs in adult age. However, it can be observed even in pediatric age, thus amplifying the clinical spectrum of this disorder, and suggesting a possible revision of the diagnostic criteria, with particular regard to the developmental age (101).

Recent research has expanded the comorbidity to several sleep disorders, such as restless legs syndrome, periodic limb movements during sleep, parasomnias, sleep-disordered breathing, and narcolepsy (82).

A detailed sleep anamnesis, relating to sleep hygiene, lifestyle habits, and the presence of sleep disorders, is mandatory in patients with headaches since it could improve the welfare of migraineur patients by correcting inappropriate sleep behaviors.

### Smoking and Headache

The correlation between smoking and headache has been hard to study in a systematic fashion (102) due to the difficulties of rigorously categorizing the type, amount, and frequency of the smoking habit. For this reason, the role of smoking in the pathogenesis or trigger role of headache is extremely controversial.

The pharmacological aspects of nicotine have been extensively studied, although they are not yet fully understood, while other toxic products of tobacco remain largely untested (103).

It is unclear whether tobacco addiction is a risk factor for headache, or headache sufferers use tobacco as an analgesic therapy for headache attacks or, at least, some kind of association between the two eventualities may be reported.

The nicotine contained in tobacco (in the form of the highly effective isomer S-nicotine) readily crosses the blood-brain barrier through passive diffusion and active transport from the choroid plexus (103). S-nicotine binds to the cortex, hippocampus, nigrostriatal, mesolimbic, thalamic, hypothalamic, midbrain, and brain stem receptors (104). Relating to some types of headache, it has been hypothesized that the Central Nervous System action of nicotine is directed in a cluster to the hypothalamus (105, 106).

Cluster Headache (CH) represents a unique feature among primary headache syndromes, due to its association with cigarette smoking or any kind of tobacco exposure (107).

Strong evidence, based on large population studies, is available to support the association between CH and smoking. In 2018 Rozen evaluated 1,134 subjects affected by cluster headache. This study observed that, in subjects who did not smoke cigarettes, the age for the onset of CH was lower and there was also a marked familiarity for CH, to suggest a greater genetic predisposition to CH. On the contrary, the later age for the CH onset in smokers could suggest the consequences of tobacco exposure and secondary toxicity from harmful metabolites (108).

Several studies have been devoted to the effect of smoking on headaches in adolescent patients.

All of them agree in defining cigarette smoking as an environmental risk factor for headache in adolescents.

In the Nord-Trøndelag Health Study, in which 8,040 adolescent students between 13 and 18 years of age underwent a self-administered questionnaire and an interview, an association between headache and smoking was reported (OR 1.5, 95% CI 1.3–1.7, *P* < 0.0001) (109).

A larger American study from the U.S. National Institute of Health, based on a group of 10.36 million students, showed that teenagers who smoked cigarettes every day exhibited symptoms such as headache, stomach ache, back pain, and morning fatigue 1.5 times more frequently than non-Smokers (110).

A more recent cross-sectional study on 5,741 students carried out in Croatia showed that teenage smokers have more frequent headaches than those who refrained from smoking (OR 1.94, CI 1.45–2.59) (111).

### Alcohol and Headache

Epidemiological studies show that more than 90% of the population had at least one episode of headache and that many have had headaches triggered by alcohol. In particular, in the work by Dueland, 75% of patients with migraines indicated alcohol consumption among the other dietary triggers, as the most frequent cause for their migraine attacks (112).

The International Classification of Headache Disorders (ICHD-3 beta revised) is an essential standpoint to classify the types of headache associated with alcohol intake. It identifies headache episodes as “secondary” when they are part of the hangover syndrome and they are not associated with any known primary headaches; they are recognized as “immediate alcohol-induced” and “delayed alcohol-induced” headaches. The first form develops within 3 h of alcohol intake; while the second form occurs after 5–12 h after drinking alcohol. Both headache forms fade out within 72 h and in both cases, they manifest characteristics different from primary headache forms (ICHD-3 beta revision) (25).

In migraineurs, on the other hand, alcohol-related headache often manifests the same features and symptoms as regular migraines. In this case, the most correct diagnosis is probably migraine or cluster headache triggered by alcohol consumption (25).

Multiple observational studies (113–116) have shown that subjects with primary headaches have a lower alcohol intake than subjects who do not suffer from headaches. Onderwatera et al. carried out a study on 1,547 patients with headaches. Close to half of the subjects were completely abstemious (much less than the general population) and just less than half of the commonly identified alcohol intake as a certain or presumed trigger for headache. The study illustrated that alcoholic drinks are commonly identified as migraine trigger factors and have an important effect on alcohol consumption behavior (117).

According to the data just described, it does not seem that alcohol may be considered the triggering factor for headache as it seems to happen in subjects with migraine and cluster headache.

Although some chemical compounds such as histamine, sulfites, tyramine, and tannins are unable to cause headache alone, they can contribute to headaches. The possibility of an inflammatory response to alcohol, triggering migraines has also been suggested (118).

In contrast to what happens in adults, in the adolescent population, the ingestion of alcohol is a risk factor for headache. In the study by Milde-Busch et al., dealing with 1,260 students aged 13–19 years and run by self-administered questionnaire and interviews, there was three times relative risk of headache disclosed among high school students in case of cocktail drinking (OR 3.4) (7).

An interesting population-based study by Arruda et al. considered the possible effects of prenatal alcohol exposure and demonstrated its association with chronic daily headaches, notably in girls, showing the correlations between headache and alcohol intake (119).

### Substances of Abuse and Headache

Substance abuse is associated with a variety of neurological complications, including headaches. Psychoactive substance abuse is growing up among adolescents in the last few decades. These substances can provoke headaches but the exact mechanisms are still unknown. In literature, there are not studies among pediatric age and what is known comes from a careful review of the literature relating to adulthood. In a large Turkish questionnaire study of 1,055 psychoactive abuse users were included and 18% of patients reported having headaches attributed to a substance or its withdrawal with a prevalence of around 30% (120). 94.5% of substance users described headaches after the onset of substance use suggesting a causal relationship. It is also reported that the substances more responsible for headache are methylamphetamine, cocaine, heroin, volatile solvent abusers (120). This large population study demonstrated that younger abusers were more inclined to primary headaches such as migraines and tension-type headaches than older users. The characteristics of headaches in abusers have reported a prevalence of bilateral location and particularly temporal ones with pulsatility (120–122).

Marinis et al. studied the relationship between headache and opiates comparing drug abusers with a control group with a higher (*p* < 0.001) incidence of headache among opiate abusers (60%), particularly those with a long history of addiction (121). The authors have reported 37.5% of patients who completed detoxification therapy configuring opiate withdrawal headache (121).

Another hypothesis was formulated by El-Mallakh et al. who analyzed self-reported questionnaires completed by 236 patients. The results showed that the onset of migraines occurred before the onset of substance use, while the onset of tension headaches occurred after the onset of substance use. Based on this data, the study proposed the probability that migraines may play a role in the genesis of substance use, while substance use may play a role in the genesis of tension headaches (123).

The pathophysiological mechanisms hypothesized underlying this type of headache may be related to chemical irritative effects on trigeminal afferents or to excitation of cortical neurons by the blockage of descending sensory inhibition, originating in the brainstem by these substances (124).

Although there are no pediatric studies in the literature, we recommend that headache patients do not take drugs because of the known complications but also because they are possible triggers of headache.

### Stress and Headache

For a long time, research has speculated that stress responses to external or internal factors may influence headaches. The origin of this belief is rooted in the neurochemical links between stress and chronic pain: on the one hand, stress produces hyperalgesia, mediated by NMDA receptors and μ receptors (125). On the other hand, it leads to a reduction of the inhibiting pain pathways (126). One more factor contributing to a chronic headache is activation, in subjects prone to chronic stress of the inflammatory process at the neuronal level, which is mediated by cytokines (127).

Acute stress is frequently reported in the literature as one of the most powerful triggers of migraine attacks in the general population. According to different studies, 50–80% of migraine episodes are caused by a stressful event both in adults (128–132) and in the pediatric population (71, 133).

Apart from being a trigger, stress has been also correlated with the development of chronic headache disease (134). De Benedittis et al. (135) retrospectively demonstrated that, in the year preceding the onset of a migraine, patients had significantly more stressful events compared to the general population. More recently, in a prospective study, Scher et al. (136) highlighted that in the general population, stressful events were more frequent in the 2 years preceding the onset of a chronic headache than in previous years. These data suggest that chronic negative events on predisposed patients could lead to migraines.

In the pediatric population, school-related stress, defined as problems with peers, teachers, and school commitments, is considered one of the primary phenomena associated with headache, (137, 138). School-related stress can also be associated with family background and adolescents with demanding parents experience a higher amount of primary headache (139).

Family background is also an important impact stressor and is frequently reported as being associated with headaches and migraines. Family dysfunctions, punitive parenting, frequent quarrels in the domestic environment, and higher parental depressive symptomatology in early life are associated with a greater likelihood of migraine in children and adolescents (140–142). In most cases in which headache is linked to comorbid stress conditions such as a dysthymic disorder (i.e., depression and bipolar syndromes), the migraine type is rarely an exclusive clinical presentation. On the contrary, a “double pattern” or even a clear picture of “tension-type headache” is often observed. Aromaa et al. (143) reported that an unhappy family atmosphere, a poorer family environment, and a bad relationship between parents are more often associated with a tension-type headache than with migraine.

Awareness that stress may have a negative impact both on the acute episodes of headache and on the severity of chronic headache disease has led to suggestions that improving the management of stress could have short and long-term positive effects on those patients. In a meta-analysis, Campbell et al. (144) showed that relaxation therapy can reduce the cephalalgia index by 33%. These results were confirmed in a more recent randomized-controlled study (145, 146). Moreover, Nestoriuc and Martin (147) conducted a meta-analytic study on the effectiveness of biofeedback in reducing the recurrence of headache attacks. These results were confirmed by Kang et al. in a successive study (148). A more detailed meta-analytic study by Hermann et al. reported that in the pediatric population, non-pharmacological prophylactic treatments such as biofeedback and relaxation therapy manifested an even more positive impact compared to the adult population: in 70% of the pediatric population studied, Hermann et al. detailed a reduction by half of the cephalalgia index (149). Therefore, it might be useful to implement good practices (relaxation therapy and biofeedback) as a prophylactic treatment for migraine in the pediatric population. This may be specifically helpful for those patients who do not manage stressful events well. This kind of intervention could also be used in association with medical treatment or the event of bad compliance, inefficacy, or contraindications for pharmacological therapies.

## Conclusions

Homeostasis and regularity are important in the pediatric population that suffers from headaches, particularly during the challenging time of childhood and adolescent life. Lifestyle recommendations should play a crucial role in the management of headache (Table 4).

**Table 4:** Lifestyle recommendations.

1. Adequate hydration: ingest 1 ounce of fluid per kilogram of body weight (89)
Expected intake of fluids: 4–8 y (all) 1.2 L
9–13 y: 1.8 L (boys); 1.6 L (girls)
14–18 y: 2.6 L (boys); 1.8 L (girls)
2. Limit or avoid the use of caffeine-drinks and cup of coffee
3. Limit or avoid the use of smoking
4. Limit or avoid the use of alcohol
5. Limit or avoid the use of videogames, watching TV, and electronic devices
6. Regular exercise: start with a 5–10 min to walk for 3/5 days a week
7. Regular sleep: at least 8 h of sleep per night are recommended
8. No skipping meals
9. Avoid the use of substances of abuse

*High consumption of cocktails (odds ratio = 3.4; 95% confidence interval 1.9–6.0) and coffee (2.4; 1.3–4.7), smoking (2.7; 1.4–5.1), and lack of physical activity (2.2; 1.3–3.7) were significantly associated with migraine plus TTH episodes, consumption of coffee, and physical inactivity particularly with migraine (3.4; 1.6–7.0; and 4.2; 2.2–7.9, respectively) and physical inactivity with TTH (1.7; 1.1–2.7). Couturier et al. (150), supposed that cerebral vasoconstriction during caffeine intake is followed by a rebound vasodilatation and increased arterial blood flow when caffeine is discontinued. Acute consumption of caffeine causes stimulation of the central nervous system, diuresis, gastric and muscle contraction, mucus secretions. It also decreased peripheral vascular resistance concomitant and increased cerebrovascular resistance and decreases smooth muscle tone, especially of the bronchial tree (27)*.

Although many studies deal with the correlation between pediatric headache and lifestyle advice, no comprehensive reviews consider all triggering factors as a whole, showing the current state of evidence. Drug treatment for acute migraine attacks is often unsatisfactory and the long-term pharmacological treatment for the prophylaxis of migraine attacks has been questioned, due to the lack of evidence on the efficacy and safety of these treatments at a developmental age. As a consequence, lifestyle recommendations play an important role in the management of this significant source of morbidity during childhood and, notably, adolescence.

Accordingly, children and teenagers with headaches should be advised to aim for a life-balance that includes regular sleep and meals, adequate hydration, adequate consumption of caffeine, reduced smoking and alcohol consumption, and regular physical activities should be promoted to prevent them from becoming overweight and to avoid stressors. An important dietary benefit for the management of migraine in adulthood may come from the ketogenic diet, which could be a new strategy in the treatment of patients of pediatric age, but further studies among the pediatric and adolescent population need to be conducted. This lifestyle advice should be considered as a first preventive treatment and be regarded as a central step in the management of programs aimed at controlling the recurrence of migraine attacks. Since headache attacks are recognized as one of the main factors influencing or even limiting the health and welfare of young people. We would expect that these educational practices be promoted with the encouragement of the International Headache Society. These lifestyle programs could promote a healthier adulthood, reducing the occasions for headache chronicity and intensifying general well-being.

## Author Contributions

UR and PP conceived and directed the review. This article's content was made by consensus of all the authors. All authors listed have made a substantial, direct and intellectual contribution to the work, and approved it for publication.

## Conflict of Interest

The authors declare that the research was conducted in the absence of any commercial or financial relationships that could be construed as a potential conflict of interest.
